# Reverse-transcription quantitative PCR directly from cells without RNA extraction and without isothermal reverse-transcription: a ‘zero-step’ RT-qPCR protocol

**DOI:** 10.1093/biomethods/bpx008

**Published:** 2017-05-30

**Authors:** Petra Chovancova, Verena Merk, Andreas Marx, Marcel Leist, Ramon Kranaster

**Affiliations:** 1 In vitro Toxicology and Biomedicine, University of Konstanz, D-78457 Konstanz, Germany; 2 Konstanz Research School Chemical Biology, University of Konstanz, D-78457 Konstanz, Germany; 3 myPOLS Biotec GmbH, D-78457 Konstanz, Germany; 4 Department of Chemistry, University of Konstanz, D-78457 Konstanz, Germany

**Keywords:** RT-qPCR, DNA polymerase, RNA, LUHMES, zero-step RT-qPCR

## Abstract

We describe an ultra-rapid and sensitive method to quantify gene expression levels in cultured cells. The procedure is based on reverse-transcription quantitative PCR (RT-qPCR) directly from cells, without RNA extraction and without an isothermal reverse-transcription step. Human neurons (Lund human mesencephalic cells) were lysed at different stages of differentiation, and the lysates were used directly as template for the combined RT-qPCR reaction. We detected a down-regulation of a proliferation marker and an up-regulation of neuronal dopaminergic genes expression. We were able to detect the reference gene target from as few as a single cell, demonstrating the application of the method for efficient amplification from small cell numbers. The data were fully in line with those obtained by the standard two-step RT-qPCR from the extracted total RNA. Our ‘zero-step’ RT-qPCR method proved to be simple and reliable with a total time from cell lysis to the end of the qPCR as short as 1.5 h. It is therefore particularly suitable for RT-qPCRs where large numbers of samples must be handled, or where data are required within short time.

## Introduction

Reverse-transcription of RNA coupled to quantification by PCR is one of the most used techniques in biological research. The reverse-transcription quantitative PCR (RT-qPCR) is usually the method of choice for rapid and sensitive quantitative measurements of mRNA copy numbers. It is used in research laboratories for gene expression analysis, e.g. for cancer phenotyping, cell or tissue response profiling, or clinical diagnosis [1–6]. To this date, the quantification of gene expression by RT-qPCR has been available via two methods: two-step RT-qPCR and one-step RT-qPCR, both involving the reverse-transcription of RNA into cDNA first, then using the cDNA as the template for qPCR amplification. In the two-step RT-qPCR, the reverse-transcription and PCR reactions are performed separately, whereas in one-step RT-qPCR they occur in the same tube. Even though the introduction of the one-step reaction reduces disadvantages of the two-step protocol, e.g. by reducing chances of contamination and pipetting errors, the method still requires the time-consuming RNA extraction and two different enzymes. The use of a reverse-transcriptase in combination with a DNA polymerase in the same tube also entails several problems that affect the efficiency of the reaction as the reagents and reaction conditions have to be adjusted for both enzymes. This impedes the use of the respective optimal conditions for the reactions [7]. Additionally, typical reverse-transcriptases are not thermostable and the reaction cannot proceed at high temperatures. Typical problems are strong secondary structures in RNA that melt only at elevated temperatures [8]. Therefore, an enzyme that can perform both reverse-transcription as well as DNA amplification would improve these drawbacks.

Recently, it has been demonstrated that reverse-transcription can be performed even under high temperatures with a novel, thermostable Taq DNA polymerase [9]. This Taq polymerase, now commercially available from myPOLS Biotec, Germany (Volcano2G DNA polymerase), has been optimized through a directed evolution approach [10] in multiple rounds of mutagenesis and screening. It combines the natural thermostability of Taq DNA polymerase with an artificially induced reverse-transcriptase activity. Therefore, RT-qPCR can be performed in a new ‘zero-step’ method—directly from RNA templates, without a need for an isothermal reverse-transcription step, as reverse-transcription takes place simultaneously with DNA amplification during the cycled PCR elongation step. The ‘zero-step’ RT-qPCR has a great potential to decrease the extent of sample handling during the reaction preparations, especially in, e.g. assays where large numbers of samples must be handled as, for instance, the characterization of markers during cellular differentiation [11–13]. However, the most time-consuming step during the RT-qPCR sample preparation is often the isolation of RNA from samples, with even the fastest RNA isolation protocols normally requiring 30–60 min of handling time for, e.g. each 10 samples. Also, availability of certain primary cells or cells from patient samples may be very low thus making it difficult to obtain sufficient RNA amounts after extraction in order to perform reliable qPCR and to quantify very low target gene copy numbers.

In neuronal as well as stem cell culture models used in basic research and toxicology, two requirements should ideally be met at the same time: a) proliferation is needed so that large numbers of cells are continuously available and b) differentiation into a stable post-mitotic state should be achieved. The Lund human mesencephalic (LUHMES) cell model meets both requirements [13, 14]. These cells, derived from primary human dopaminergic cells, were conditionally immortalized by introducing a tetracycline responsive *v-myc* gene [15] allowing a proliferating culture and maintenance similar to other cell lines. The v-myc gene expression can be switched off to arrest the growth and trigger a homogeneous differentiation of the cells to a dopaminergic phenotype. LUHMES phenotype and function as a neuronal model have been characterized [13] and several dopaminergic and neuronal gene markers were identified as up- or down-regulated during stages of the cells’ differentiation into mature dopaminergic neurons.

In this study, we used this well-characterized cell differentiation model and studied the expression of marker genes during the transition of these neuronal precursor cells into mature dopaminergic neurons. We show that the novel ‘zero-step’ RT-qPCR performed well without RNA isolation, directly from cell samples, following a simple and fast protocol with standard cycling times. Compared with a conventional two-step RT-qPCR assay with a DNA-polymerase-Sso7d fusion protein, which is leading to increased processivity and reduced reaction times, the simplified direct-from-cells ‘zero-step’ RT-qPCR produces faster and highly reliable results, while minimizing potential errors and reducing reagents expenditure.

## Materials and methods

### LUHMES culture

LUHMES cells (ATCC® CRL-2927™) were cultured exactly as described earlier [13]. Briefly, the proliferating culture was maintained in Nunclon™ flasks (Thermo Fisher Scientific, USA) coated with 50 µg/ml poly-l-ornithine and 10 µg/ml fibronectin (Sigma Aldrich, Germany) in Advanced Dulbecco’s modified Eagle’s medium/F12, supplemented with 1× N-2 supplement (Invitrogen, Germany), 2 mM l-glutamine (Gibco, Germany), and 40 ng/ml basic fibroblast growth factor (bFGF) (R&D Systems, The Netherlands). For differentiation, 150 000 cells/cm^2^ cells were seeded in Advanced Dulbecco’s modified Eagle’s medium/F12, supplemented with 1× N-2 supplement (Invitrogen), 2 mM l-glutamine (Gibco), 1 mM dibutyryl cyclic adenosine monophosphate (cAMP) (Sigma Aldrich), 10 µg/ml tetracycline (Sigma Aldrich), and 2 ng/ml human glial cell-derived neurotrophic factor (GDNF) (R&D Systems).

### Immunocytochemistry

LUHMES cells, cultured and differentiated on pre-coated glass bottom 8-well µ-slides (Ibidi, Germany) at cell density of 150 000 cells/cm^2^, were fixed with 4% paraformaldehyde (Sigma Aldrich) for 15 min at RT, washed and permeabilized with 0.2% Triton X-100 in phosphate buffered saline (PBS) for 10 min at RT. Blocking solution of 5% bovine serum albumin (Calbiochem, USA) was then added for 1 h at RT. Mouse anti-TUJ1 primary antibody (Covance, USA) diluted 1:500 was then added overnight at 4 °C. Samples were washed three times with PBS/0.05% Tween and anti-mouse Alexa-488 secondary antibody (Invitrogen) were applied for 1 h at RT in dark. 1 µg/ml Hoechst-33342 (Molecular Probes, USA) was added 10 min before the incubation with the secondary antibody was over. Cells were then washed three times with PBS and imaged with LSM 880 confocal point laser scanning microscope equipped with a GaAsP detector (Zeiss, Germany) using a 40× oil objective. Image processing was carried out with the Fiji software.

### Cell dilution preparation

1.5 × 10^6^ d0 LUHMES cells were lysed in 1 ml of VolcanoCell2G lysis buffer (myPOLS Biotec) for 15 min on ice and transferred into Eppendorf tube. From this initial stock, a dilution series of decreasing numbers of cells was prepared in the lysis buffer so that 2 µl of a dilution added to a reaction tube corresponded to the desired cell number to be analyzed (1–3000 cells). The prepared cell lysates were stored at −80 °C until RT-qPCR reaction run.

### RNA extraction, reverse-transcription, qPCR (two-step); and RT-qPCR (zero-step)

For a two-step RT-qPCR, RNA was extracted at corresponding time points using PureLink RNA Mini Kit (Thermo Fisher Scientific). The total RNA amount was quantified using Nanodrop (Thermo Fisher Scientific). 1 µg RNA was firstly primed for 5 min at 25 °C, then reverse-transcribed using iScript™ Reverse Transcription Supermix (Bio-Rad, US) for 30 min at 42 °C and the reverse-transcriptase was inactivated for 5 min at 85 °C. The subsequent cDNA was diluted 1:10 in nuclease-free water and stored at −20 °C. For the qPCR, reaction mixtures (10 µl) contained 5 µl of SsoFast™ EvaGreen® Supermix (Bio-Rad), 0.4 µM of the respective forward and reverse primers, and 2 µl of the thawn cDNA. After an initial denaturation cycle (98 °C for 2 min) the product was amplified in 40 PCR cycles (98 °C for 2 s, 60 °C for 5 s) followed by a melting curve analysis using the Roche LightCycler® 96 System (Roche, Switzerland).

For a ‘zero-step’ RT-qPCR, cells were lyzed directly in the culture well using VolcanoCell2G Lysis Buffer (myPOLS Biotec) for 15 min at 4 °C. Cell lysate dilutions were prepared in the lysis buffer and stored at −80 °C. Thawed diluted supernatant from approximately 1500 cells was then used as a template for the RT-qPCR reaction. Reaction mixtures (10 µl) contained 5 µl of VolcanoCell2G 2× RT-PCR Master Mix (myPOLS Biotec), 0.1 µM of the respective hydrolysis probe, and 0.4 µM of the respective forward and reverse primers. The amount of template in each reaction was equivalent to the amount of mRNA in approximately 1500 cells. After an initial denaturation step (95 °C for 3 min) the product was amplified in 40 PCR cycles (95 °C for 15 s, 62 °C for 75 s). Real-time quantification was performed using hydrolysis probes (TaqMan™ probes). A detailed protocol for the ‘zero-step’ RT-qPCR is provided in the Supplementary Material.

In general, all qPCR reactions for each target gene were set up manually in triplicates of three biological replicates (three independent cell differentiations) from day 0 to day 8 LUHMES. Primers, probes, and their targets are described in the Supplementary Material.

The quantification cycles (Cq) were analyzed for each gene and gene expression levels were presented as relative expression compared with the reference gene glyceraldehyde-3-phosphate dehydrogenase (*GAPDH*) (2^−^^(ΔCq)^). ΔCq = Cq(day X, gene Y) – Cq(day X, *GAPDH*). The data were analyzed with GraphPad Prism 5.0.

## Results and discussion

### Phenotypic changes during neuronal differentiation

**Figure 1:**
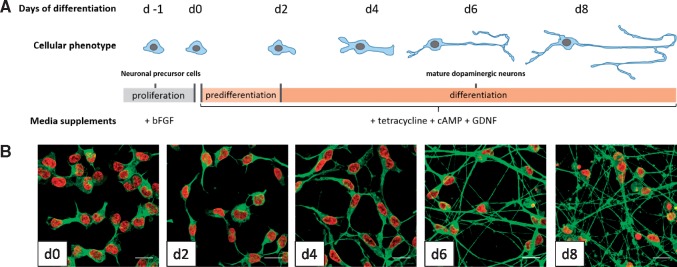
Differentiation of LUHMES cells. (**A**) Schematic representation of the differentiation procedure showing phenotypic changes during the differentiation from neuronal precursor cells to mature dopaminergic neurons. (**B**) Representative fluorescent confocal microscopy images of LUHMES immunostained during different stages of maturation (d0–d8 of differentiation) for βIII-tubulin (green). Nuclei are labeled by DNA staining with H-33341 dye (red). Scale bar = 20 µm.

For a rough control of correct differentiation of neuronal precursors into mature dopaminergic neurons, we observed the phenotypic changes of the cells. The differentiation of LUHMES was initiated by depleting the cells of bFGF, culturing them in medium supplemented with tetracycline, cAMP, and GDNF (Fig. 1A) as previously described [13]. The immunostaining for βIII-tubulin, a protein primarily expressed in neurons [16], showed that undifferentiated precursor cells on day 0 (d0) did not display any neurites. The neurite growth was then observed from day 2 (d2) on. It progressed throughout the differentiation stages. On day 6 of the differentiation (d6), the culture consisted of uniformly post-mitotic and mature neurons, which showed an elaborate network of elongated neurites (Fig. 1B). Phenotypically, the differentiation had proceeded as expected.

### Expression of neuronal markers during differentiation by ‘zero-step’ RT-qPCR directly from cells

**Figure 2:**
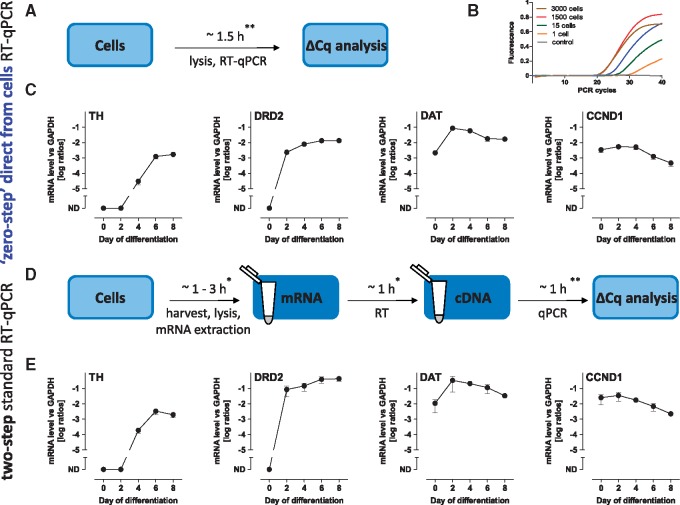
Comparison of neuronal marker expression via ‘zero-step’ direct-from-cells and two-step RT-qPCR methods. (**A**) Experimental setup of the ‘zero-step’ direct-from-cells RT-qPCR without RNA extraction and without separate isothermal reverse-transcription steps. (**B**) Amplification curves of *GAPDH* gene from a dilution series samples of LUHMES cells (0–3000 cells). (**C**) mRNA expression levels of *TH, DRD2, DAT1*, and *CCND1* during LUHMES differentiation (d0–d8) determined by ‘zero-step’ RT-qPCR using VolcanoCell2G Master Mix. (**D**) Experimental setup of a standard two-step RT-qPCR including the RNA extraction, reverse-transcription (RT), and qPCR steps. (**E**) mRNA expression levels of *TH, DRD2, DAT1*, and *CCND1* during LUHMES differentiation (d0–d8) determined by two-step RT-qPCR using iScript™ Reverse Transcription Supermix and SsoFast™ EvaGreen® Supermix. Data are means ± SD of three independent LUHMES differentiations, each consisting of three technical replicates normalized to *GAPDH* expression (*TH*, tyrosine hydroxylase; *DRD2*, dopamine receptor D2; *DAT*, dopamine transporter; *CCND1*, cyclin D1). ND = not detectable. *, these durations were estimated based on ∼45 samples and can vary due to the method of choice for mRNA purification. **, these durations were estimated based on the preparation and run of one 96-well RT-qPCR plate.

To demonstrate the differentiation of LUHMES cells along the dopaminergic lineage on a transcriptional level, and to test the ability of VolcanoCell2G DNA polymerase to facilitate ‘zero-step’ RT-qPCR directly from cells, we first defined the ideal cell number needed for the reaction setup. Undifferentiated LUHMES cells were lysed for 15 min in VolcanoCell2G Lysis Buffer on ice, and dilutions of 3000, 1500, 150, 15, and 1 cell were prepared. These were used as a template for the ‘zero-step’ RT-qPCR reaction (Fig. 2A) to amplify *GAPDH* as a reference gene. The target was detectable even with the lysate corresponding to as little as one cell per reaction with increasing signal in an increasing-cell-amount manner. The exception was an amplification from 3000 cells, where lower fluorescence was detected. This may have been a consequence of cellular debris quenching the fluorescent signal during the qPCR or high amount of cells in the template leading to an inhibition of the qPCR reaction. The highest signal and the lowest quantification cycle (Cq) value were measured from the reaction containing 1500 cells (Fig. 2B). This was therefore considered to be an ideal cell amount for the template, and it was selected for amplification of further targets in order to detect changes in mRNA expression levels of different markers during neuronal cell maturation.

Several markers can be tested to confirm the extent of differentiation of LUHMES cells along the dopaminergic lineage [13]. Here, we looked at the expression of tyrosine hydroxylase (*TH*)—one of the most important markers of mature dopaminergic neurons, the presynaptic dopamine receptor D2 (*DRD2*), dopamine transporter (*DAT*), and cyclin D1 (*CCND1*)—the regulator of the cell cycle progression. To perform RT-qPCR directly from cells, these were lysed during different stages of neuronal maturation (day 0–day 8) in the culture well by an addition of VolcanoCell2G Lysis Buffer and pelleted by centrifugation. The amount of the lysate’s supernatant corresponding to 1500 cells was then added directly into the reaction mix containing VolcanoCell2G Master Mix, as well as primers and RT-qPCR was performed. The transcript levels of *TH* were not detectable until day 4 of differentiation, then rising rapidly on day 6. As expected, *DRD2* was detectable from day 2, reaching its maximum level on day 6. *DAT* was maximally up-regulated on day 2 and then slightly decreased in expression. These three markers confirm the differentiation of LUHMES along the dopaminergic lineage. The down-regulation of *CCND1* further confirmed the proliferation arrest and cellular differentiation into mature neurons by day 6 (Fig. 2C). Thus, we confirmed the successful differentiation of LUHMES cells along the dopaminergic lineage using the RT-qPCR method directly from cells. VolcanoCell2G allowed extremely easy and fast handling of the samples in only 15 min, without any need for RNA purification. This reduced not only the bench time, but also prevented any potential sample loss or mix-up. For this experiment, cells were grown in different dishes and at different stages of maturation. Such complex starting conditions are often encountered in assays involving differentiation of, e.g. precursor cells, stem cells, toxicity studies, or high-throughput assays, where multiple samples must be handled in the same experiment. Compared with a two-step protocol, which involves multiple pipetting steps from cell culture to the ready-to-use RNA sample, the ‘zero-step’ protocol reduces these to a single pipetting step, therefore lowering the risk of potential errors. Moreover, the optimal cell amount for the ‘zero-step’ RT-qPCR in case of LUHMES was 1500 cells which is very low and shows that the ‘zero-step’ RT-qPCR may prove to be useful for assays, where numbers of cells are very limited such as, for instance, patient’s samples or primary cells.

### Comparison to a conventional two-step RT-qPCR method

To further evaluate the performance of the ‘zero-step’ direct-from-cells RT-qPCR, we compared it with a standard two-step RT-qPCR assay (Fig. 2D). The differentiating d0–d8 cells were lysed in a standard commercial lysis buffer and RNA was extracted from the lysates. Afterwards, the RNA amount was measured and reverse-transcription performed to obtain cDNA from the samples. This was used as a template for the qPCR reaction with the qPCR master mix and primers in the reaction mix. The results were very similar to the ones obtained with the ‘zero-step’ RT-qPCR, showing the same patterns of up-regulation of *TH, DRD2*, and *DAT* and down-regulation of *CCND1* during the progression of neuronal differentiation (Fig. 2E). While both RT-qPCR methods showed the same pattern of up and down-regulation, we observed a lower standard deviation of the Cq values within replicates when using VolcanoCell2G, which in turn resulted in statistically more significant data (Supplementary Materials 3 and 4). The observed lower reliability of the data obtained with the two-step RT-qPCR may be explained by the multiple handling and pipetting of the samples during the RNA extraction, subsequent dilution, and/or cDNA preparation, whereas with the ‘zero-step’ RT-qPCR these steps were omitted. We also noted that the apparent mRNA levels were higher in the two-step RT-qPCR assay, a phenomenon often observed when comparing different qPCR assays [17]. One reason may be due to the fact that the used iScript™ reverse-transcriptase has Ribonuclease H (RNase H) activity, removing remaining RNA that is present after the mRNA has been reverse-transcribed [18]. VolcanoCell2G DNA polymerase on the contrary is lacking any RNAase activity. Another explanation for the differences between the ‘zero-step’ and two-step RT-qPCR’s detected mRNA amounts may also be accounted to the fact, that in the two-step RT-qPCR assay, a double-stranded DNA (dsDNA) binding dye was used. Usage of this dye (EvaGreen®) may lead to an earlier detectable fluorescent signal than a probe-based assay, which is used for the ‘zero-step’ assay. A dsDNA binding dye can also incorporate into non-specific dsDNA which may generate false positive signals, whereas probe-based assays are known to be more specific and generate fluorescent signals only after significant production of the specific amplified complementary sequences. As the up- and down-regulation patterns in the expression of the detected target genes were identical in both, ‘zero-step’ as well as the two-step RT-qPCR assays, we propose that the ‘zero-step’ RT-qPCR produces reliable results, however in an improved time and cost-efficient manner.

We demonstrate here a fast RT-qPCR method that can be performed directly from cell lysates. The results obtained from reactions performed directly from cell lysates with the ‘zero-step’ protocol were approximately equivalent to those obtained from purified RNA that was reverse-transcribed and then amplified in the two-step protocol. Our data therefore show that the ‘zero-step’ direct-from-cell RT-qPCR performs just as well as the standard method, while saving consumables as well as handling and bench time. Therefore, the ‘zero-step RT-qPCR’ protocol has the potential to become an important technique in cell line screening for specific target genes. The method allows researcher to gain a fast and reliable overview of the cellular mRNA targets by eliminating time-consuming and error-prone intermediate steps.

## Authors’ contributions

P.C. and V.M. contributed equally to the study, performed the experiments, and wrote the manuscript. R.K., A.M., and M.L. critically revised the data and manuscript.

*Conflict of interest statement.* R.K. and A.M. are co-founders and employees of myPOLS Biotec GmbH.

## Supplementary Material

Supplementary Data
