# Supplementary material for: Reverse-transcription quantitative PCR directly from cells without RNA extraction and without isothermal reverse-transcription: a ‘zero-step’ RT-qPCR protocol
Source: Biol Methods Protoc. 2017 May 30;2(1):bpx008. doi: 10.1093/biomethods/bpx008 (PMC6977950; doi:10.1093/biomethods/bpx008)
Supplement: Supplementary Data [file bpx008_supp.zip › Chovancova_et_al_Suppl_2_Oligos.doc]

Supplemetary Material 2

Primers and probes information for:

**Reverse-transcription quantitative PCR directly from cells without RNA extraction and without isothermal reverse-transcription: a ‘zero-step’ RT-qPCR protocol**

*Petra Chovancova ^1,2^, Verena Merk ^4^, Andreas Marx ^3,4^, Marcel Leist ^1^ and Ramon Kranaster ^4*^*

^1^	In vitro Toxicology and Biomedicine, University of Konstanz, D-78457 Konstanz, Germany;

^2^	Konstanz Research School Chemical Biology, University of Konstanz, Germany;

^3^	Department of Chemistry, University of Konstanz, D-78457 Konstanz, Germany;

^4^	myPOLS Biotec GmbH, D-78457 Konstanz, Germany

All primers and probes were designed with the “RealTime qPCR Tool“, which can be found on the homepage from Integrated DNA technologies: <http://eu.idtdna.com/site>.

All sequences 5’ to 3’:

**CCND1: cyclin D1**

[NM_053056.2](https://www.ncbi.nlm.nih.gov/entrez/viewer.fcgi?db=nucleotide&id=77628152) Homo sapiens cyclin D1 (CCND1), mRNA

```
product length = 225
Forward primer  1    CATCTACACCGACAACTCCATC  22
Template        584  ......................  605
Reverse primer  1    GGCGGATTGGAAATGAACTTC  21
Template        808  .....................  788

Probe:         Fam – CGCAATGACCCCGCACGATTTC – BMNQ535
```

**DAT: Dopamine transporter**

[NM_001044.4](https://www.ncbi.nlm.nih.gov/entrez/viewer.fcgi?db=nucleotide&id=262399362) Homo sapiens solute carrier family 6 member 3 (SLC6A3), mRNA

```
product length = 172
Forward primer  1    TCAACGACACTTTTGGGACC  20
Template        737  ....................  756
Reverse primer  1    GTCTTCACGCCCTTCCAG  18
Template        908  ..................  891

Probe: Fam – CAGCACGCCACGTTCAAAGTACTC - BMNQ535
```

**DRD2: dopamine receptor D2**

[XM_017017296.1](https://www.ncbi.nlm.nih.gov/entrez/viewer.fcgi?db=nucleotide&id=1034572280) PREDICTED: Homo sapiens dopamine receptor D2 (DRD2), transcript variant X1, mRNA

```
product length = 245
Forward primer  1    TGGTAGGTGAGTGGAAATTCAG  22
Template        504  ......................  525
Reverse primer  1    TGCGTTATTGAGTCCGAAGAG  21
Template        748  .....................  728
```

[NM_016574.3](https://www.ncbi.nlm.nih.gov/entrez/viewer.fcgi?db=nucleotide&id=181337009) Homo sapiens dopamine receptor D2 (DRD2), transcript variant 2, mRNA

```
product length = 245
Forward primer  1    TGGTAGGTGAGTGGAAATTCAG  22
Template        522  ......................  543
Reverse primer  1    TGCGTTATTGAGTCCGAAGAG  21
Template        766  .....................  746
```

[NM_000795.3](https://www.ncbi.nlm.nih.gov/entrez/viewer.fcgi?db=nucleotide&id=181336946) Homo sapiens dopamine receptor D2 (DRD2), transcript variant 1, mRNA

```
product length = 245
Forward primer  1    TGGTAGGTGAGTGGAAATTCAG  22
Template        522  ......................  543
Reverse primer  1    TGCGTTATTGAGTCCGAAGAG  21
Template        766  .....................  746

Probe: Fam – AGTGGGCAGGAGATGGTGAAGG - BMNQ535
```

**GAPDH: Homo sapiens glyceraldehyde-3-phosphate**

[NM_001289746.1](https://www.ncbi.nlm.nih.gov/entrez/viewer.fcgi?db=nucleotide&id=576583523) Homo sapiens glyceraldehyde-3-phosphate dehydrogenase (GAPDH), transcript variant 4, mRNA

```
product length = 176
Forward primer  1    ACATCGCTCAGACACCATG  19
Template        159  ...................  177
Reverse primer  1    CATGGGTGGAATCATATTGGAAC  23
Template        334  .......................  312
```

[NM_001289745.1](https://www.ncbi.nlm.nih.gov/entrez/viewer.fcgi?db=nucleotide&id=576583518) Homo sapiens glyceraldehyde-3-phosphate dehydrogenase (GAPDH), transcript variant 3, mRNA

```
product length = 176
Forward primer  1    ACATCGCTCAGACACCATG  19
Template        265  ...................  283
Reverse primer  1    CATGGGTGGAATCATATTGGAAC  23
Template        440  .......................  418
```

[NM_002046.5](https://www.ncbi.nlm.nih.gov/entrez/viewer.fcgi?db=nucleotide&id=576583510) Homo sapiens glyceraldehyde-3-phosphate dehydrogenase (GAPDH), transcript variant 1, mRNA

```
product length = 176
Forward primer  1    ACATCGCTCAGACACCATG  19
Template        173  ...................  191
Reverse primer  1    CATGGGTGGAATCATATTGGAAC  23
Template        348  .......................  326

Probe:  Cyanine 5 – AAGGTCGGAGTCAACGGATTTGGTC – BMNQ650
```

**TH: Tyrosine hydroxylase**

[XM_011520335.2](https://www.ncbi.nlm.nih.gov/entrez/viewer.fcgi?db=nucleotide&id=1034575132) PREDICTED: Homo sapiens tyrosine hydroxylase (TH), transcript variant X1, mRNA

```
product length = 159
Forward primer  1     CCGTGCTAAACCTGCTCTTC  20
Template        2546  ....................  2565
Reverse primer  1     GCACGAAGTACTCCAGGTG  19
Template        2704  ...................  2686
```

[NM_199293.2](https://www.ncbi.nlm.nih.gov/entrez/viewer.fcgi?db=nucleotide&id=88900504) Homo sapiens tyrosine hydroxylase (TH), transcript variant 3, mRNA

```
product length = 159
Forward primer  1    CCGTGCTAAACCTGCTCTTC  20
Template        339  ....................  358
Reverse primer  1    GCACGAAGTACTCCAGGTG  19
Template        497  ...................  479
```

[NM_000360.3](https://www.ncbi.nlm.nih.gov/entrez/viewer.fcgi?db=nucleotide&id=88900502) Homo sapiens tyrosine hydroxylase (TH), transcript variant 2, mRNA

```
product length = 159
Forward primer  1    CCGTGCTAAACCTGCTCTTC  20
Template        258  ....................  277
Reverse primer  1    GCACGAAGTACTCCAGGTG  19
Template        416  ...................  398
```

[NM_199292.2](https://www.ncbi.nlm.nih.gov/entrez/viewer.fcgi?db=nucleotide&id=88900500) Homo sapiens tyrosine hydroxylase (TH), transcript variant 1, mRNA

```
product length = 159
Forward primer  1    CCGTGCTAAACCTGCTCTTC  20
Template        351  ....................  370
Reverse primer  1    GCACGAAGTACTCCAGGTG  19
Template        509  ...................  491

Probe: Fam – TGTCCCGAGCTGTGAAGGTGTTT - BMNQ535
```
