# Supplementary material for: Reverse-transcription quantitative PCR directly from cells without RNA extraction and without isothermal reverse-transcription: a ‘zero-step’ RT-qPCR protocol
Source: Biol Methods Protoc. 2017 May 30;2(1):bpx008. doi: 10.1093/biomethods/bpx008 (PMC6977950; doi:10.1093/biomethods/bpx008)
Supplement: Supplementary Data [file bpx008_supp.zip › Chovancova_et_al_Suppl_1_Protocol.doc]

Supplementary Material 1

Protocol for:

**Reverse-transcription quantitative PCR directly from cells without RNA extraction and without isothermal reverse transcription: a ‘zero-step’ RT-qPCR protocol**

*Petra Chovancova ^1,2^, Verena Merk ^4^, Andreas Marx ^3,4^, Marcel Leist ^1^ and Ramon Kranaster ^4*^*

^1^	In vitro Toxicology and Biomedicine, University of Konstanz, D-78457 Konstanz, Germany;

^2^	Konstanz Research School Chemical Biology, University of Konstanz; Germany;

^3^	Department of Chemistry, University of Konstanz, D-78457 Konstanz, Germany;

^4^	myPOLS Biotec GmbH, D-78457 Konstanz, Germany

**REAGENTS**

VolcanoCell 2G RT-PCR 2x Master Mix (myPOLS Biotec, Germany)

50x VolcanoCell 2G Lysis Buffer (myPOLS Biotec, Germany), consisting of (Tris pH 9.2 (2.5 M), (NH_4_)_2_SO_4_ (800 mM), MgCl_2_ (175 mM), proprietary mix of detergents (30 %)).

Nuclease-free water

Primers (binding on exon-exon junctions)

Hydrolysis probes

**Instrumentation:** PCR cycler (e.g. Roche LightCycler® 96 System) (Roche, Switzerland)

**PROCEDURE**

**Cell lysis and dilution**

*Attention:* Keep samples on ice at all time during the lysis procedure.

1. Seed/ treat cells in cell-culture flasks, dishes or well-plates (for adherent cultures) or roller bottles (for suspension cultures).

**For adherent cultures:**

2. Remove media and place culture plate/flask on ice.
3. Add VolcanoCell2G Lysis Buffer directly to the plate. 100 µl Lysis Buffer is recommended for 100,000 cells, which corresponds to a final amount of 1000 cells/µl.
4. Incubate on ice for 15 min and shake the plate occasionally.

*Attention:* Some very adherent cell types may remain adhered to the culture plastic/glass. Do not scrape them as only the supernatant is needed for the reaction setup.

5. Pellet any remaining cell debris by transferring the supernatant from the culture flask/well-plate into a tube and centrifuging at >10,000 rpm for 1 min at room temperature.
6. Prepare a dilution series of the cell lysis supernatant corresponding to 1 - 5000 cells in VolcanoCell2G Lysis Buffer in order to determine the best amount of cells for the PCR reaction. This needs to be done once for each different cell type.

*Rest:* The prepared PCR template (cell lysate supernatant) can be stored at -80°C.

*Attention:* Make the dilutions in VolcanoCell2G Lysis Buffer. Keep samples during all handling steps at 4°C.

**For suspension cell cultures:**

7. Pellet cells by centrifugation and remove the supernatant.
8. On ice, add the VolcanoCell2G Lysis Buffer (100 µl Lysis Buffer is recommended for 100,000 cells which corresponds to a final amount of 1000 cells/µl) and resuspend the cell pellet by gently pipetting up and down.
9. Incubate for 15 min on ice and shake the tube occasionally.
10. Centrifuge at 10,000 rpm for 1 min at room temperature to pellet cell debris.
11. Prepare a dilution series of the cell lysis supernatant corresponding to 1 - 5000 cells in order to determine the best amount of cells for the PCR reaction.

*Rest:* The prepared PCR template (cell lysate supernatant) can be stored at -80°C. *Attention:* Prepare the dilutions in VolcanoCell2G Lysis Buffer. Keep samples during all handling steps at 4°C.

**‘Zero-step’ RT-q**PCR reaction setup

| **Component** | **20 µl reaction** | **Final concentration** |
| --- | --- | --- |
| VolcanoCell2G RT-PCR 2x Master Mix | 10 µl | 1x |
| Primer forward (10 µM) | 0.8 µl | 0.4 µM (0.05 - 1 µM) |
| Primer reverse (10 µM) | 0.8 µl | 0.4 µM (0.05 - 1 µM) |
| Probe (2.5 µM) | 0.8 µl | 0.1 µM |
| Template/Cell suspension | 1 - 6 µl | between 1 - 5000 cells/rxn |
| Nuclease-free water | Up to 20 µl |  |

**‘Zero-step’ RT-qPCR protocol**

*Attention:* No isothermal reverse-transcription step is needed

25 - 40

cycles

| **Step** | **Temperature** | **Time** |
| --- | --- | --- |
| Initial denaturation | 95°C | 3 min |
| Denaturation | 95°C | 15 sec |
| Annealing/Extension* | 55 - 75°C | 60 sec – 75 sec |
| Hold | <10°C | hold |

* The annealing temperature is usually 3 - 5°C below the calculated melting temperature of the primers used. Ideally, establish the PCR by running a temperature gradient in order to find the best annealing/extension temperature for each primer pair.

**TROUBLESHOOTING**

**No amplification detectable**

**Problem:** Unspecific product formation

**Solutions:**

- Keep all reagents during PCR setup on ice.

- Program the PCR protocol on the cycler first, before setting up the PCR mix and if applicable preheat the lid of the cycler.

- When establishing a new PCR assay, start with a typical standard protocol and recommended reaction setup concentrations. Run a temperature gradient to identify the best temperature yielding in the cleanest product. This temperature is only limited by the primer annealing temperature, as VolcanoCell2G polymerase is thermostable.

- Use a "multiple primer analyzer" to test primer sequences for self-complementarity, secondary structures and primer cross annealing.

- Try a different set of primers for the target
